# Serine/Threonine Protein Kinase SpkG Is a Candidate for High Salt Resistance in the Unicellular Cyanobacterium *Synechocystis* sp. PCC 6803

**DOI:** 10.1371/journal.pone.0018718

**Published:** 2011-05-26

**Authors:** Chengwei Liang, Xiaowen Zhang, Xiaoyuan Chi, Xiangyu Guan, Youxun Li, Song Qin, Hong bo Shao

**Affiliations:** 1 Qingdao University of Science and Technology, Qingdao, China; 2 Institute of Oceanology, Chinese Academy of Sciences, Qingdao, China; 3 Yellow Sea Fisheries Research Institute, Chinese Academy of Fishery Sciences, Qingdao, China; 4 Ocean University of China, Qingdao, China; 5 Yantai Institute of Coastal Zone Research, Chinese Academy of Sciences, Yantai, China; University of South Florida, United States of America

## Abstract

**Background:**

Seven serine/threonine kinase genes have been predicted in unicellular cyanobacterium *Synechocystis* sp. PCC6803. SpkA and SpkB were shown to be required for cell motility and SpkE has no kinase activity. There is no report whether the other four STKs are involved in stress-mediated signaling in *Synechocystis* PCC6803.

**Methodology/Principal Findings:**

In this paper, we examined differential expression of the other four serine/threonine kinases, *SpkC*, *SpkD*, *SpkF* and *SpkG*, at seven different stress conditions. The transcriptional level was up-regulated of *spkG* and down-regulated of *spkC* under high salt stress condition. Two *spk* deletion mutants, ΔspkC and ΔspkG, were constructed and their growth characteristic were examined compared to the wild strain. The wild strain and ΔspkC mutant were not affected under high salt stress conditions. In contrast, growth of *spkG* mutant was completely impaired. To further confirm the function of *spkG*, we also examined the effect of mutation of *spkG* on the expression of salt stress-inducible genes. We compared genome-wide patterns of transcription between wild-type *Synechocystis* sp. PCC6803 and cells with a mutation in the *SpkG* with DNA microarray analysis.

**Conclusion:**

In this study, we first study the *spkG* gene as sensor of high salt signal. We consider that *SpkG* play essential roles in *Synechocystis* sp. for sensing the high salt signal directly, rather than mediating signals among other kinases. Our microarray experiment may help select relatively significant genes for further research on mechanisms of signal transduction of *Synechocystis* sp. PCC6803 under high salt stress.

## Introduction

Cyanobacteria are photoautotrophic prokaryotes able to grow in a wide range of ecological environments, so their signal transduction systems, which perceive and transduce environmental signals, are important in the acclimation to the environmental changes. In prokaryotes, one-component and two-component signal transduction systems are the pre-eminent mechanisms for signal transduction [1], [2], [3]. In contrast, the Ser/Thr-specific protein kinases (STK) serve as the backbone of the eukaryotes transduction network [4]. However, with the first identification of an STK in *Myxococcus xanthus* in 1991 [5], regulatory STKs have been repeatedly identified in prokaryotes, and some of them have been shown to regulate various cellular functions, such as development [6], [7], [8], stress responses [9], [10] and pathogenicity [11]. Protein phosphorylation on serine/threonine residues in cyanobacteria was first revealed by radioactive labeling of proteins in 1994 [12]. Now, a large number of genes are emerging with the availability of whole genome sequences. A total of 286 putative STK genes have been identified from 21 species of cyanobacteria, and functions of most of them are still unknown [13]. The entire nucleotide sequence of the *Synechocystis* sp. PCC6803, a strain bearing the ability to be transformed naturally [14], was the first cyanobacterial genome to be sequenced. Genome sequence data of *Synechocystis* has predicted the presence of 7 serine/threonine kinase (PKN2 type) genes, named *spkA* (*sll1574–1575*), *spkB* (*slr1697*), *spkC* (*slr0599*), *spkD* (*sll0776*), *spkE* (*slr1443*),* spkF* (*slr1225*) and *spkG* [15]. Six of them have been biochemically characterized with the exception of *SpkG* which was not expressed in *Escherichia coli*. [15], [16], [17]. SpkA, SpkB, SpkC, SpkD and SpkF were demonstrated as autophosphorylation and phosphorylation of the general substrate proteins. SpkE did not show any protein kinase activities, which was consistent with its lack of several key amino acid residues. But only SpkA and SpkB were confirmed to be essential for the motility of *Synechocystis* cells [15], [16]. In high plant, environmental stresses trigger a wide variety of plant responses, ranging from altered gene expression and cellular metabolism to changes in growth rate and plant productivity [18]. Here, we examined probable physiological roles of SpkC, SpkD, SpkF and SpkG in *Synechocystis* . To investigate whether the four STKs are involved in stress-mediated signaling in *Synechocystis* PCC6803, the expression of *spkC*, *spkD*, *spkF* and *spkG* were examined under a variety of stress conditions by semi-quantitative RT-PCR. We found the expression of *spkC* was down-regulated but expression level of *spkG* was increased to a significantly extent at high salt conditions. In order to better understand the function of *spkG* and *spkC* and their importance in salt stress, we constructed a knock-out mutant and compared the feature of this strain to that of the wild-type in the presence and absence of high salt 855 mM NaCl (0.5 g/l NaCl). The growth of the *spkG* mutant strain was completely impaired at 855 mM NaCl conditions in contrast to the wild strain and ΔspkC mutant, which were unaffected. Based on the above results, the microarrays were used for a high-precision look at differential gene expression when comparing wild-type to a mutant or when comparing differently environmental conditions. We obtained the regulatory genes under high salt stress were induced or repressed by spkG.

## Results and Discussion

### Semi-quantitative reverse transcription PCR

**Figure 1:**
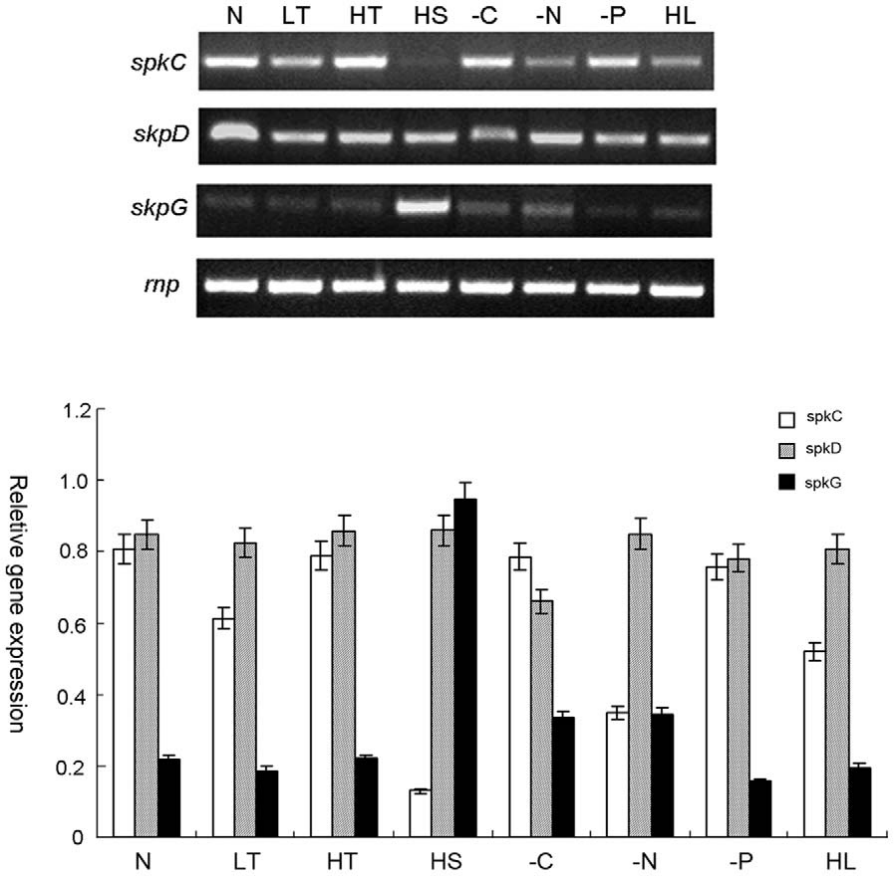
Semiquantitative RT-PCR reveals the transcriptional dynamics of spkC, spkD, spkF and spkG at different conditions. The transcript level of RNase P in each sample serves as a control. N, normal conditions; LT, low temperature; HT, high temperature; HS, high salt; −C, carbon-deficient; −N, nitrogen-deficient; −P, phosphorus-deficient. The normal cultivation condition and all stress conditions have been detailed in Materials and methods.

To investigate whether the four STKs are involved in stress-mediated signaling in *Synechocystis* PCC6803, the expression of *spkC*, *spkD*, *spkF* and *spkG* were examined under a variety of stress conditions by semi-quantitative RT-PCR. Total RNA isolated from *Synechocystis* PCC6803 untreated and stress-treated cells was used to amplify *spkC*, *spkD*, *spkF* and *spkG* cDNA. The amplified RT-PCR fragment was confirmed by cloning and sequencing. Semi-quantitative RT-PCR expression analysis showed that under standard conditions, *spkC* and *spkD* showed relatively high levels compared to *spkG* (Fig. 1), and there was no detective expression of *spkF*. A severe decrease was observed in mRNA accumulation for *spkC* after the treatment of high salt stress and a moderate decrease was detected after the treatment of nitrogen deprivation and high light stress. The levels of *spkC* transcripts under other stress conditions did not vary significantly. This suggested that the expression of *spkC* were down-regulated under high salt conditions. The expression levels of *spkD* did not vary significantly under normal and different stress conditions but was relatively higher compared to the others, consistent with the fact that it is essential for survival,which could not be knocked out completely [17]. No expression of *spkF* could be detected under any condition (data not shown). After exposure to high salt stress, the expression level of *spkG* was increased to a significantly extent. A densitometry measurement showed that the induced level of *spkG* transcripts s about 1.3±0.02-fold higher than the constitutive level(Fig. 1), while the other stress conditions had no effect on the transcription level. The up-regulated expression of *spkG* suggested a mechanism of positive regulation under high salt stress. In two-component signal transduction systems, most histidine kinases are known to respond to stress by post-translational alterations and regulatory proteins could be affected at both the transcriptional and post-transcriptional levels [19]–[22]. Also, in Rice, calmodulin-dependent serine/threonine phosphatase was reported to be involved in the process of salt stress tolerance [23]. We examined the transcriptional levels of serine/threonine kinases using RT-PCR and our results suggested that, in *Synechocystis* at least, STKs could respond to stress conditions by controlling their expression levels.

### The detection of mutants and growth curve

**Figure 2:**
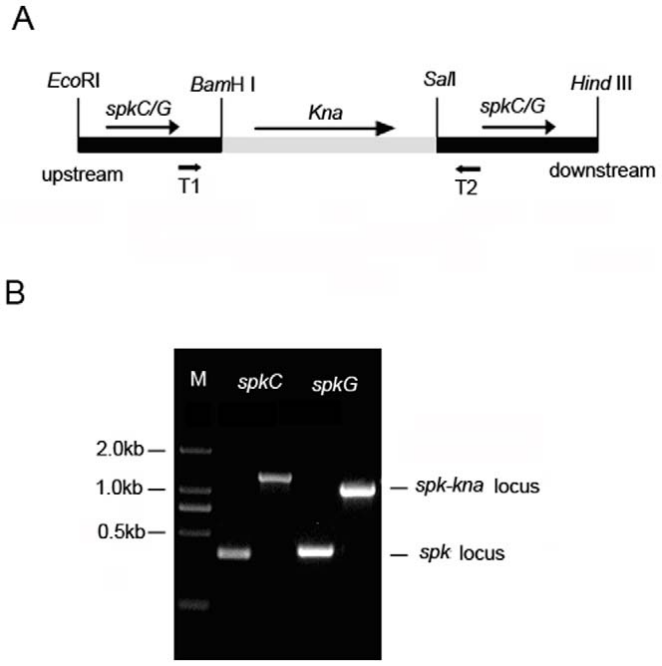
Insertional mutagenesis of the spkC and spkG genes in *Synechocystis* PCC6803. (A) Schematic representation of the constructs used to generate ΔspkC and ΔspkG mutants. The above arrows indicate directions of transcription. The restrictive sites were all added at the end of the primers, as shown in Table 1. Primers of T1 and T2, same with those for RT-PCR, are used for PCR determination of completely mutant separation. (B) PCR determination of the complete separation of ΔspkC and ΔspkG mutants. M, DL2000 marker.

**Figure 3:**
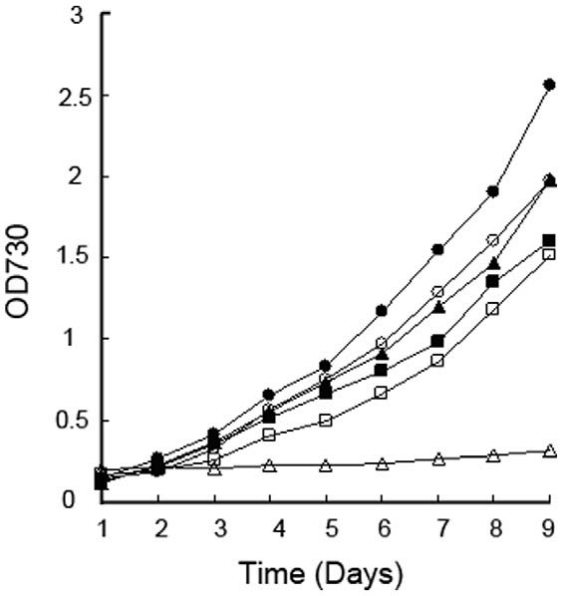
Growth properties of wild strain (circles) and ΔspkC (rectangles) and ΔspkG (triangles) mutants at normal (filled symbols) and high salt (empty symbols) stress conditions. To determine the reliability of these data, each experiment was performed three times. Data are from one representative experiment.

To further affirm the probable functions of *SpkC* and *SpkG* in high salt stress, two mutants, ΔspkC and ΔspkG, were generated by replacing part sequences of these two genes with kanamycin resistance cassettes (Fig. 2A) and the complete segregation of mutants were verified by PCR analysis (Fig. 2B). We examined the growth of ΔspkC and ΔspkG mutants under standard and high salt-stress conditions compared to that of the wild strain. The growth properties of the two mutants were not significant changed compared to the wild strain under normal conditions. The presence of 684 mM NaCl, the same condition with RT-PCR, in the growth medium has no effect on the growth of ΔspkC, ΔspkG and wild strain (data not shown). Whereas, the growth of the ΔspkG mutant strain was almost completely impaired at 855 mM NaCl conditions in contrast to the wild strain and ΔspkC mutant, which were unaffected (Fig. 3). During their long evolution, cyanobacteria have adapted to aquatic habitats with various salt concentrations [24]. *Synechocystis* sp. PCC6803 has the ability to regulate essential metabolic processes to enable survival in high salt environments [25]. This result indicates that *SpkG* may be involved in adaptation to high salt stress condition. In our previous work, we predicted that SpkG has four strong transmembrane helices by the TMbase (www.ch.embnet.org/software/TMPRED_form.html) [13]. So we predicted that *SpkG* could sense the high salt signal directly, rather than mediate signals among other kinases.

### Examination of the genome-wide expression of high salt-stress genes

**Figure 4:**
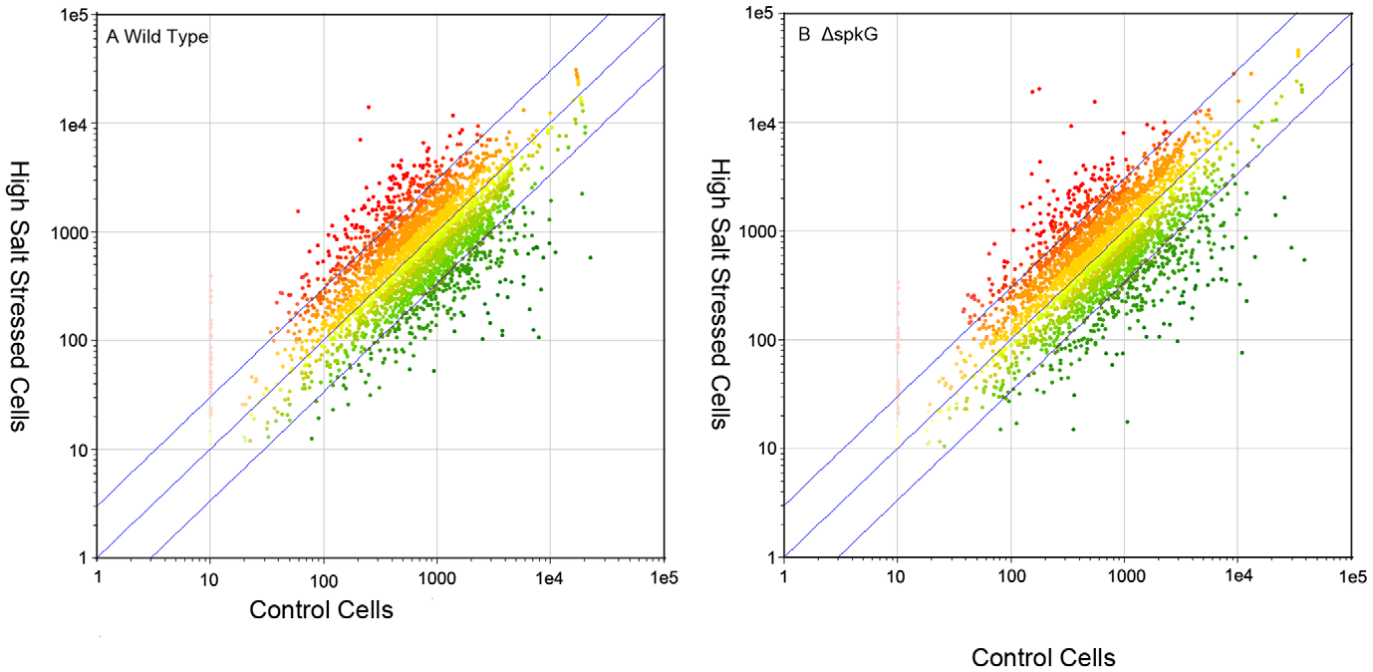
Genome-wild patterns of transcription of salt stress regulated genes. A. Gene expression in wild-type cells that had been exposed to 855 mM NaCl for 30 min was compared with that in unstressed cells. B. Gene expression in ΔspkG cells that had been exposed to 855 mM NaCl for 30 min was compared with that in unstressed cells. Dots correspond to genes whose fold change >2 are beyond the reference lines.

To elucidate the effect of *spkG* on the adaptation to high salt condition, we used DNA microarrays to investigate gene expression of salt-stress *Synechocystis*, wild types of cells and ΔspkG mutants were treated with high salt-stress at 855 mM NaCl conditions for 30 min. The results of the first screening were confirmed by a second screening. The raw data of microarray in this study was released in a MIAME compliant database with accession number A-MEXP-1953. The results are summarized in Fig. 4. The average signal intensities of treated samples are plotted versus average signal intensities of control samples and the two reference lines are corresponding to ratios of expression of 2.0 and 0.5 in Figure 4. The putative differentially expressed genes were finally selected based on the expression profiles and the following criteria: the data pointing outside this range were regarded as genes that are salt stress-inducible or repressible genes in our DNA microarray experiments. Thus, approximately 25% of the genes in *Synechocystis* responded positively to high salt stress. 401 genes were induced more than twofold and 358 genes were repressed more than twofold after high salt-stress for wild type *Synechocystis*, while 401 genes were induced more than twofold and 240 genes were repressed more than twofold for ΔspkG mutants.

To identify genes whose expression is affected by spkG upon exposure of cells to high salt stress, we compared genome-wide patterns of transcription between wild-type and ΔspkG cells. The putative differentially expressed genes were finally selected based on the expression profiles and the following criteria: if the average fold change between wild type and mutants cells was more than or equal to twofold. Sixty genes have been found expressed differently in wild strain and ΔspkG mutant, the genes that could be divided into four groups: (i) Group 1, genes that were normally salt-regulated genes was no longer regulated by salt stress genes in ΔspkG mutants. (ii) Group 2, genes whose salt inducibility was moderately diminished in ΔspkG mutants. (iii) Group 3, genes whose salt regulation was heightened in ΔspkG mutants. (iv) Group4, genes were regulated by salt stress only in ΔspkG mutants (Table S1).

### Gene whose expression was induced or repressed by salt-stress was regulated by spkG

Using a DNA microarray, we compared the genome-wide patterns of transcription in wild-type and ΔspkG cells upon high salt stress to demonstrate the effect of the mutation in *spkG* on the high salt regulation genes. We found expression of 15 of the normally salt-regulated genes was no longer regulated by salt stress in ΔspkG mutants (Table S1, group 1). Many of the genes whose salt inducibility were diminished by mutation of spkG. It appeared that the regulation of expression of particular sets of genes by high salt in wild type was controlled by *spkG*. *spkG* was essential for the regulation of the expression of 15 genes (group 1) and was partially involved in the regulation of 12 genes (group 2). This group includes genes for proteins whose functions are related to transporters and mechanisms. nrtC and nrtD were reported as a member of the ABC (ATP-binding cassette) superfamily of active transporters. It is essential for the growth of the cyanobacterium at physiological concentrations of nitrate and has been shown to be involved in the active transport of nitrite as well [26] OprB is considered a central component of carbohydrate transport and described as a carbohydrate-selective porin [27]. The ferric uptake regulator (Fur) protein, as originally described in *Escherichia coli*, is a global regulator that acts as a transcriptional repressor when it binds ferrous ion. Fur also activates the expression of many genes by either indirect or direct mechanisms [28].

### Genes whose salt stress-regulated expression were not regulated by spkG

The salt inducibility of a large group of genes was unaffected by mutations in *spkG*, shows the functionally characterized and most strongly salt-induced members of this group. In particular, the fourth group of genes was regulated by salt stress only in ΔspkG mutants. This group included the following genes: plsC for 1-acyl-sn-glycerol-3-phosphate acyltransferase; amiA for N-acetylmuramoyl-L-alanine amidase Enzyme catalyzing the preferential transfer of unsaturated fatty acids to the 2-position of the glycerol backbone of phospholipids and triacylglycerols ; ndhA for NADH dehydrogenase subunit I; a Trans-Isoprenyl Diphosphate Synthases Squalene/phytoene synthase(*sll0513*) synthesis; a C-methyltransferase(*slr1610*) and a number of other genes for protein with known and unknown function. It is possible that there are several sensory mechanisms in the perception of high salt stress. When the presence of *spkG*, spkG protein could sense the high salt signal directly and regulated some genes to response to high salt. However, when the absence of *spkG*, an additional mechanism was activated. Hik16, Hik33, Hik34 and Hik41, have been reported to respond to salt stress and regulate the expression of only one-fifth of the salt-inducible genes [29]. The induction of the remaining 80% salt-inducible genes was unaffected by any histidine kinases mutants, suggesting an additional mechanism absent in *Synechocystis* 6803. However, the mechanisms involved in sensing specific salt stress signals are not well resolved [20]. Thus, it is probable that complex mechanisms involved in the regulation of the response to salt stress remain to be identified and characterized. In the future, more analysis will be necessary for defining the functions of the many unknown proteins regulated in salt stress cells, which will help us to identify the complex salt-sensing mechanisms in *Synechocystis* sp. PCC6803.

## Materials and Methods

### Strains and growth conditions

The *Synechocystis* sp. PCC6803 strain was kindly provided by Pro. Xudong Xu (Institute of Hydrobiology, Chinese Academy of Science). Wild-type and derived mutant cells of *Synechocystis* sp. PCC6803 were grown photoautotrophically in BG11 medium [30] at 30°C and 30 µmol m^−2^ s^−1^ white light. Solid BG11medium was supplemented with 1% agar, 8 mM TES (PH8.2) and 5 mM sodium thiosulfate. For mutants, kanamycin was added to a final concentration of 30 µg/ml in the solid medium and 50 µg/ml in the liquid medium. Cells grew to mid-logarithmic growth phase (optical density at 730 nm of 0.6∼0.8) before different stress treatment. For high light treatment, cells were diluted with fresh medium to an OD730 of ∼0.3, and then were exposed to high light (500 µmol m^−2^ s^−1^) for 30 min. For temperature shock experiments, cells were shifted to 15°C and 42°C for 30 min separately. For high salt stress, cells were exposure to 684 mM NaCl for 30 min. In the nutrient deprivation experiment, cells were cultivated in BG-11 medium lacking carbon (−C), nitrogen (−N), or phosphorus (−P) for 2 h. Growth characteristics under high salt stress were examined in BG11 medium with 684 mM and 855 mM NaCl.

### Isolation of total RNA

RNA was isolated from 50 ml cultures of wild-type cells. The cells were collected by centrifugation (5000 g for 5 min) and the cell pellets were cooled in liquid nitrogen. Then cooled cells were grinded into powder under the liquid nitrogen and were resuspended in 1 ml Biozol reagent and Chloroform (ratio of 1∶5 to Biozol). An equal volume of isopropyl alcohol were added to the aqueous phase after centrifuged at 12000 g for 15 min at 4°C, and the solution was incubated for 2 h at −20°C. Precipitated RNA was collected by centrifugation at 12000 g for 15 min at 4°C and washed with 70% ethanol, resuspended in DEPC-H_2_O. The total RNA was treated with RNase-free DNase I (Promega) at 37°C for 30 min to remove DNA.

### RT-PCR analysis

RT-PCR primers were designed to amplify 350–400 bp of internal coding region of each gene (Table 1). Reverse transcription reactions were performed with the random hexamer primers using M-MLV Reverse Transcriptase (Promega). The resulting cDNA was used as the template for RT-PCR. Amplified products were electrophoretically examined on 1% agarose gels. The transcript abundance of rnpA (*slr1469*) encoding RNase P were used as external standards.

**Table 1:** Oligonucleotide sequences used in this study.

Primer	Sequences(5′-3′)	Restrictive sites
Mutant construction		
spkC(*slr0599*)		
Upstream	TTGAATTCGTTACCCCACTCAAAC	*EcoR*I
	ATGGATCCCTTGGTCTATGACCTT	*BamH*I
Downstream	ATGTCGACTTGCTAATGGCAAGAC	*Sal*I
	AATAAGCTTCCCTAATTTTGCTCGG	*Hind*III
spkG(*slr0152*)		
Upstream	CAGAATTCATGGTAAAGACTGCCA	*EcoR*I
	TAGGATCCCTTTGGGGTAATTTCT	*BamH*I
Downstream	ACGTCGACAAGTAAATCAAGACCA	*Sal*I
	ACCAAGCTTAATGTTTTCCACTTGG	*Hind*III
kna	TCGGATCCAGTCAGCAACCATAGT	*BamH*I
	ATGTCGACCCGCTCAGAAGAACT	*Sal*I
RT-PCR		
spkC(*slr0599*)	CAGTTTGGGACTAACGGC	
	TAAACCTTGGTGGCTTGG	
spkD(*sll0776*)	CACTAGGGGATTTATGG	
	TTGGTGGAACTTCTCGT	
spkF(*slr1225*)	TCTTAGTTTCGTCCACGG	
	ACCCTGATTGCCTTTACC	
spkG(*slr0152*)	TTACTTGCCCCCATTGT	
	CTCCCTGGATTAAGAGGC	
rnp(*slr1469*)	GGACTACCCAAAACACTGC	
	CAATAATCCCAGCTTGGCT	

### DNA manipulation and mutant construction and culvation

To inactivate spkC gene, two fragments (upstream and downstream) were separately amplified by PCR. Each primer was added a different restriction site at the 5′ end (Table 1). These two fragments were then cloned into the pMD-18T vector (TAKARA) one by one after double-digested, resulting in plasmid pMD-CUD. The plasmid pEGFP-1 (BD) was used as template to amplify the selectable cassette encoding the *kna* genes for resistance to kanamycin. This 1.0 kb *kna* fragment was then cloned into plasmid pMD-CUD via *BamH*I and *Sal*I sites added at the ends of the primers, yielding plasmid pCUKD. The plasmid pGUKD to inactive *spkG* gene was constructed with the same method. Cells of *Synechocytis* sp. PCC6803 were transformed with plasmids pCUKD and pGUKD according to Williams [31]. Transformants were selected by screening for resistance to 30 µg of kanamycin/ml in BG-11 solid medium supplemented with 10 mM glucose. Segregation of the inactivated *spkC* and *spkG* genes were monitored by PCR using genomic DNA of transformants used as the template and primers that recognize sequences upstream or downstream of the inserted *kna* gene.

### MicroArray analysis

A *Synechocystis* DNA microarray (CyanoCHIP) was purchased from TaKaRa Bio Co. Ltd (Kyoto). This microarray covered 3,079 of the 3,168 ORFs of *Synechocystis*, excluding genes for transposases. As hybridization probes, Cy3 dyelabeled or Cy5 dyelabeled cDNAs were used, which were synthesized by reverse transcription of total RNA using an RNA Fluorescence Labeling kit (MMLV) provided by TaKaRa. The method of hybridization was performed as described previously [32]. Hybridization of the dye-labeled cDNAs was evaluated with an array scanner. For quantification with the IIlumina BeadArry Reader, the local background of each spot was subtracted and the signal was normalized by transforming it to the ratio of the spot-specific intensity relative to the total intensity of signals from all genes with the exception of rDNA genes. Therefore, changes in the level of transcript of each gene relative to the total level of mRNAs were calculated. Each gene is spotted twice on the microarray, allowing signal evaluation and error exclusion. The gene expression in cells exposed for 30 min to 855 mM NaCl was analyzed by two DNA-microarray experiments. All data is MIAME compliant and the raw data has been deposited in a MIAME compliant database.

## Supporting Information

Table S1 **Influence of high salt on levels of transcripts in wild-type cells and the ΔspkG mutant.**
(DOC)
