# Supplementary material for: Serine/Threonine Protein Kinase SpkG Is a Candidate for High Salt Resistance in the Unicellular Cyanobacterium Synechocystis sp. PCC 6803
Source: PLoS One. 2011 May 26;6(5):e18718. doi: 10.1371/journal.pone.0018718 (PMC3102658; doi:10.1371/journal.pone.0018718)
Supplement: Table S1 — Influence of high salt on levels of transcripts in wild-type cells and the ΔspkG mutant. (DOC) [file pone.0018718.s001.doc]

Table S1. Influence of high salt on levels of transcripts in wild-type cells and the ΔspkG mutant*a*

| **Gene** | **putative function** | **Wild type** | **ΔspkG** |
| --- | --- | --- | --- |
| **Group1：** | | | |
| slr0019 | unknown | 3.023±0.355 | 1.349±0.023 |
| slr1903 | transposase | 2.977±0.829 | 1.351±0.119 |
| sll0994 | function unknown | 0.350±0.133 | 0.944±0.232 |
| slr0042 oprB | Carbohydrate-selective porin | 0.122±0.013 | 0.938±0.032 |
| sll1680 | methionine sulfoxide reductase B | 0.463±0.03 | 0.94±0.152 |
| slr0044(nrtD) | nitrate transport protein NrtD | 0.291±0.075 | 1.072±0.299 |
| slr1513 | unknown | 0.299±0.077 | 0.995±0.006 |
| sll0218 | unknown | 0.32±0.093 | 1.135±0.473 |
| slr0043(nrtC) | nitrate transport protein NrtC | 0.326±0.092 | 1.260±0.04 |
| slr0095 | O-methyltransferase | 0.454±0.023 | 0.885±0.209 |
| sll1784 | unknown | 0.459±0.024 | 0.837±0.031 |
| slr1704 | unknown | 0.407±0.047 | 0.816±0.149 |
| sll0567(fur) | ferric uptake regulation protein | 0.386±0.087 | 0.79±0.066 |
| sll1188 | hypothetical protein unknown | 0.371±0.037 | 0.736±0.04 |
| sll0622(nadA) | quinolinate synthetase | 0.389±0.725 | 0.744±0.077 |
| **Group2：** | | | |
| slr1805 | sensory transduction histidine kinase | 0.331±0.053 | 0.542±0.129 |
| slr0709YjgH | hypothetical protein | 0.312±0.014 | 0.6±0.021 |
| sll0846 | unknown | 0.033±0.002 | 0.061±0.010 |
| slr0967 | pentapeptide | 0.068±0.116 | 0.188±0.043 |
| sll1726 | unknown | 5.170±0.175 | 3.397±0.602 |
| slr1703(serS) | seryl-tRNA synthetase | 5.186±0.235 | 2.780±0.605 |
| slr0817(entC) | isochorismate synthase | 6.693±1.441 | 3.946±0.772 |
| slr1638 | unknown | 4.400±0.792 | 2.646±0.016 |
| slr0712 | Predicted integral membrane protein | 7.860±0.281 | 4.501±0.091 |
| sll0854 | unknown | 4.409±0.061 | 2.747±0.231 |
| sll1716 | Transposase | 7.430±1.498 | 2.827±0.363 |
| sll1473 | GAF PAS | 5.688±0.054 | 2.717±0.128 |
| slr1245 | transcriptional regulator | 5.205±0.808 | 2.377±0.640 |
| slr0899(cynS) | Cyanatelyase, Cyanase | 2.343±0.333 | 0.583±0.121 |
| **Group4：** | | | |
| slr2103 plsC | 1-acyl-sn-glycerol-3-phosphate acyltransferase | 1.52±0.220 | 10.275±0.375 |
| ssr2315 | unknown | 1.869±0.086 | 3.495±1.200 |
| slr1956 | TPR | 1.057±0.016 | 2.856±0.279 |
| slr2006 | unknown | 1.468±0.428 | 2.965±0.117 |
| slr1279(ndhC) | NADH dehydrogenase subunit 3 | 1.248±0.250 | 2.660±0.564 |
| sll1340 | unknown | 1.372±0.420 | 2.612±0.272 |
| slr0516 | pentapeptide | 1.176±0.189 | 3.297±0.887 |
| slr1826 | unknown | 1.661±0.215 | 6.441±3.409 |
| slr0254 | RDD | 1.648±0.085 | 3.600±0.136 |
| slr1910(amiA) | N-acetylmuramoyl-L-alanine amidase | 1.831±0.765 | 4.027±1.501 |
| sll0519(ndhA) | NADH dehydrogenase subunit 1 | 1.674±0.496 | 4.072±1.519 |
| sll0513 | Trans-Isoprenyl Diphosphate Synthases Squalene/phytoene synthase | 0.988±0.217 | 2.244±0.202 |
| slr1610 | C-methyltransferase | 1.023±0.354 | 2.519±0.428 |
| slr0251 | ABC transporter | 1.380±0.340 | 3.072±0.073 |
| sll0517(rbpA) | RNA binding protein | 1.246±0.189 | 0.296±0.039 |
| slr1512 | unknown | 0.427±0.033 | 1.732±0.123 |
| **Group5：** | | | |
| slr1046 | unknown | 2.124±0.153 | 7.75±1.150 |
| slr0747(msiK) | ATP binding protein responsible for multiple sugar import | 3.575±0.626 | 7.225±0.425 |
| slr1222 | unknown | 4.549±1.120 | 16.750±1.650 |
| slr0492(menE) | O-succinylbenzoic acid—CoA ligase | 2.509±0.485 | 14.575±3.325 |
| slr1623 | unknown | 2.410±0.210 | 4.484±0.853 |
| slr1626 | Hypothetical protein | 2.474±0.473 | 6.825±0.875 |
| ssr3570 | unknown | 4.378±1.147 | 7.950±0.650 |
| slr0023 | unknown | 4.536±1.388 | 18.725±2.325 |
| sll0703 | unknown | 3.154±0.444 | 8.475±1.025 |
| sll0436 | unknown | 2.567±0.003 | 4.228±0.153 |
| slr0252 | precorrin-6x reductase | 7.289±0.086 | 21.915±7.835 |
| sll0983 | unknown | 2.502±0.374 | 11.688±5.542 |
| sll0030 LysR | LysR substrate binding domain | 4.754±0.696 | 8.425±0.575 |
| sll1515 | unknown | 0.538±0.456 | 0.166±0.001 |
| sll1874(AT103) | phytochrome-regulated gene | 0.255±0.045 | 0.095±0.011 |
